# Involvement of free radicals in breast cancer

**DOI:** 10.1186/2193-1801-2-404

**Published:** 2013-08-27

**Authors:** Sandra Ríos-Arrabal, Francisco Artacho-Cordón, Josefa León, Elisa Román-Marinetto, María del Mar Salinas-Asensio, Irene Calvente, Maria Isabel Núñez

**Affiliations:** Departamento de Radiología y Medicina Física, Universidad de Granada, Av. Madrid s/n, 18012 Granada, Spain; Instituto de Investigación Biosanitaria de Granada, Granada, Spain; Ciber de Enfermedades Hepáticas y Digestivas CIBERehd, Granada, Spain; Instituto de Biopatología y Medicina Regenerativa (IBIMER), Universidad de Granada, Av. Conocimiento, s/n, 18100 Armilla Granada, Spain

## Abstract

Researchers have recently shown an increased interest in free radicals and their role in the tumor microenvironment. Free radicals are molecules with high instability and reactivity due to the presence of an odd number of electrons in the outermost orbit of their atoms. Free radicals include reactive oxygen and nitrogen species, which are key players in the initiation and progression of tumor cells and enhance their metastatic potential. In fact, they are now considered a hallmark of cancer.

However, both reactive species may contribute to improve the outcomes of radiotherapy in cancer patients. Besides, high levels of reactive oxygen species may be indicators of genotoxic damage in non-irradiated normal tissues. The purpose of this article is to review recent research on free radicals and carcinogenesis in order to understand the pathways that contribute to tumor malignancy.

This review outlines the involvement of free radicals in relevant cellular events, including their effects on genetic instability through (growth factors and tumor suppressor genes, their enhancement of mitogenic signals, and their participation in cell remodeling, proliferation, senescence, apoptosis, and autophagy processes; the possible relationship between free radicals and inflammation is also explored. This knowledge is crucial for evaluating the relevance of free radicals as therapeutic targets in cancer.

**Electronic supplementary material:**

The online version of this article (doi:10.1186/2193-1801-2-404) contains supplementary material, which is available to authorized users.

## Introduction

The role of free radicals in the genesis of different diseases has been widely documented (Okezie et al. 1991; Polidori et al. 2001; Jomova & Valko 2011). Besides having specific cell functions, they can become toxic for the cells that produce them or for neighboring cells in contact in a tissue or organ. This is the case of oxygen, a highly stable molecule, which can turn into different reactive species, some with the character of free radicals, after participating in some cell metabolism functions. These free radicals constitute the product or are used to perform important cell functions, especially when the reactivity of molecular oxygen is insufficient (Turi et al. 2002).

The cell generates free radicals and also degrades that which is strictly necessary to avoid the damage derived from a non-controlled formation. However, various intrinsic and extrinsic circumstances and the biochemical activity of the cell can make it lose control over the formation and management of free radicals. This imbalance in the formation and use of free radicals in tissue is known as “oxidative stress”. It results from a disturbance of the balance between the formation of reactive oxygen species (ROS) and the defense provided by cell antioxidants (Schafer & Buettner 2001; Shen et al. 2011). This imbalance may cause damage related to various human diseases (Polidori et al. 2001). Hence, the term “oxidative stress-related diseases” is already in use, understood as clearly defined functional or pathological anomalies that involve the participation of free radicals (Mena et al. 2009; Jomova & Valko 2011). This imbalance can also be affected by interaction with metals, including iron (Skrzydlewska et al. 2005; Iolascon et al. 2009), copper (Prousek 2007; Speisky et al. 2009), chromium (Quievryn et al. 2002; Reynolds et al. 2007), and cobalt (Pourahmad et al. 2003; Kim et al. 2008), producing symptomatic effects in numerous diseases (Valko et al. 2006). Cancers, numerous inflammatory processes that lead to cancer, and some autoimmune diseases, have been attributed to the direct or indirect effect of free radical-induced oxidative stress (Turi et al. 2002). The application of chemotherapy and radiotherapy in cancer treatments can also favor oxidative stress (Halliwell & Gutteridge 2007; Panis et al. 2012).

### Formation of free radicals

Free radicals are highly unstable and reactive due to the presence of an odd number of electrons in the outermost orbit of their atoms; their aggressive action derives from their attempts to attain “balance” by binding with electrons of neighboring atoms, giving rise to chain reactions (Halliwell 1999; Griendling et al. 2000).

**Figure 1:**
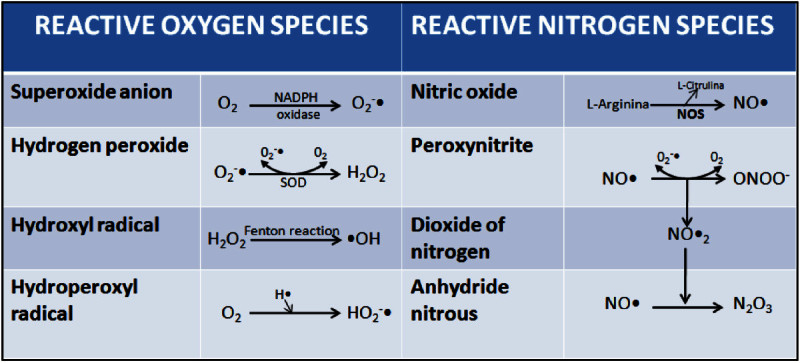
**Representative diagram of the different oxygen and nitrogen reactive species.**

The superoxide free radical (O_2_^-^•) is formed by action of the NADPH oxidase enzyme, and is most abundantly produced the mitochondrion (Cadenas & Sies 1998). Subsequently, the highly reactive hydrogen peroxide (H_2_O_2_) is formed by the action of superoxide dismutase (SOD) in a process known as dismutation, which takes place when the superoxide radical reacts with itself and forms oxygen and hydrogen peroxide (Griendling et al. 2000). A further reduction process transforms the hydrogen peroxide *via* the Fenton reaction into the hydroxyl free radical (•OH), and finally, water is formed as the final product, mediated by the action of catalase (CAT) or glutathione peroxidase (GPx) (Griendling et al. 2000; Jomova & Valko 2011). Furthermore, in cases in which an oxygen molecule binds to a proton, another free radical is formed known as the hydroperoxide radical (HO_2_^-^•) (Cuzzocrea et al. 2001). The different reactive forms of oxygen are designated ROS (Figure 1) (Griendling et al. 2000; Jian Liu & Rosenberg 2005; Valko et al. 2007).

Reactive nitrogen species (RNS) (Figure 1) include the free radical nitric oxide (NO•), peroxynitrite (ONOO^-^), the radical nitrogen dioxide (NO_2_•) and nitrite (NO_2_^-^) (Mahelkova et al. 2008). NO• is synthesized from a guanidine group of L-arginine by an enzyme of the nitric oxide synthetase (NOS) family. The formation of ONOO^-^ takes place by the reaction of NO• with a molecule of O_2_^-^•, which yields nitrogen dioxide (NO_2_•) as intermediary. This intermediary reacts with NO• to finally generate anhydride nitrous (N_2_O_3_) (Ridnour et al. 2004; Cuzzocrea et al. 2001; Valko et al. 2007).

The different isoforms of NOS include: neuronal (nNOS or NOSI), inducible (iNOS or NOSII), endothelial (eNOS or NOSIII), and mitochondrial (mtNOS) forms, all dependent on NADPH and calmodulin (Singh & Gupta 2011). eNOS is important in tumor development because it modulates various cancer-related processes, such as apoptosis, angiogenesis, cell cycle, invasion, and metastasis (Dudzinski & Michel 2007). Many of the biological actions of NO• are mediated by guanylyl cyclase (sGC) and cyclic guanylate monosphosphate (cGMP). NO• spreads to adjacent cells and readily enters the cytosol, where it activates sGC by binding to the “hemo” component of iron on the porphyrin ring. At high concentrations, its cytotoxic effects inhibit mitochondrial enzymes, including succinate and ubiquinone oxidoreductase, and aconitase, which are important in cell metabolism (Nathan 1992; Cuzzocrea et al. 2001).

### Site of production and function of free radicals

Various cellular metabolic systems constantly produce free radicals from oxygen. Thus, 80% of molecular oxygen is consumed in mitochondria, and 5% of this is transformed into superoxide and hydroxyl radicals. Endogenous (prostaglandins, fatty acids, etc.) and exogenous (drugs, colorants, flavorings, antioxidants, etc.) substances are metabolized in the smooth endoplasmic reticulum, consuming 15% of molecular oxygen, of which 20-30% is reported to be transformed into free radicals, especially •OH.

Macrophages and leucocytes, as defense mechanisms against bacteria and virus, also contribute to the formation of free radicals. Free radicals are used in prostaglandin synthesis, as in the synthesis of cholesterol and steroidal hormones. The hydroxylation of lysine and proline amino acids to hydroxylysine and hydroxyproline, respectively, necessary for collagen biosynthesis, requires the participation of hydroxyl free radicals (Wright et al. 1994).

Hence, free radicals have an essential function in the normal metabolism of cells. However, their presence poses a risk, especially for large molecules, e.g., nucleic acids, proteins, polymerized carbohydrates (polysaccharides), and lipids, which are preferentially damaged by oxygenated free radicals; (Davies & Delsignore 1987; Wright et al. 1994; Ferrari et al. 2009; Ziech et al. 2010).

### Defense of the organism against free radicals

Antioxidants are the first source of protection of the body against free radicals and other oxidants, being the compounds that halt the attack and the formation of radical species within cells. The group of antioxidants inside the organism is known as the total antioxidant state (TAS) (Teixeira et al. 2013), which is responsible for transforming free radicals into molecules that are less damaging for the organism. Examples of these antioxidants are peroxisomal catalase (CAT), SOD, and GPx (Jones et al. 2000). Glutathione is also an important antioxidant compound responsible for maintaining intracellular redox homeostasis. This redox balance is altered under hypoxia conditions, as in the case of tumors, with the production of ROS and NO•. Glutathione exists in reduced (GSH) and oxidized (glutathione disulphide, GSSG) states. In its reduced state, it sequestrates ROS, which is transformed and recycled by the action of the glutathione-reductase enzyme (GRd). The electron source used by this enzyme is coenzyme NADPH, which mainly derives from the phosphate pentose pathway. GSH is an essential cofactor for antioxidant cells known as GSH peroxidases, including GPx, which are used to detoxify peroxides, including the H_2_O_2_ generated in cell membranes that react with GSH. H_2_O_2_ have a double role in carcinogenesis, as explained further on (Brigelius-Flohé & Kipp 2009). In healthy cells and tissues, over 90% of total glutathione is in reduced form GSH and less than 10% in disulphide form GSSG (Griffith 1999). An increase in the proportion of GSSG to GSH is considered to indicate oxidative stress (Martínez Sarrasague et al. 2006; Valko et al. 2007; Badjatia et al. 2010; Delwar et al. 2011).

### Free radicals in carcinogenesis

ROS and RNS contribute in different ways to carcinogenesis and the malignant progression of tumor cells, enhancing their metastatic potential. In fact, they are now considered a distinctive characteristic of cancer. These species lead to genomic damage and genetic instability, and they participate as intermediaries in mitogenic and survival signals *via* growth factor receptors and adhesion molecules, promoting cell mobility, inducing inflammation/repair and angiogenesis in the tumor microenvironment (Klaunig & Kamendulis 2004; Valko et al. 2006; Valko et al. 2007; Roberts et al. 2009; Pani et al. 2010; Klaunig et al. 2010; Pande et al. 2011; Fuchs-Tarlovsky 2013; Pervin et al. 2013).

### Free radicals and damage in macromolecules: genetic instability

Free radicals may produce breaks and considerable damage in the DNA molecule, producing mutations and eventually cancer. The main source of mutations in live organisms is DNA damage by oxidation, with an estimated frequency of 10^4^ lesions/cell/day in human cells (Coussens & Werb 2002; Klaunig et al. 2010). The amino acids that form proteins may also undergo alterations that modify their molecular structure, hindering their biological action. In the case of enzymes, oxidative damage may hinder their catalytic action. Polysaccharides, which play a part in epithelium protection and/or lubrication roles, may also be affected, thereby reducing defenses and favoring inflammations (Zaremba & Olinski 2010).

Lipids, especially those containing polyunsaturated fatty acids, are especially prone to non-controlled oxidation induced by free radicals. They produce major damage in cell membranes, where these fatty acids have an essential function. The phrase “we age because we oxidize” has been cited by various authors over many years (Wright et al. 1994). There is evidence that oxidation products accumulate in aged individuals. The model of damage to purine and pyrimidine bases suggests that at least part of the damage is inflicted by •OH, suggesting that •OH is formed in the nucleus. ROS/RNS can also damage mitochondrial DNA, and this damage acquires importance in both human diseases and the aging process (Cuzzocrea et al. 2001).

### Free radicals as mitogenic signal intermediaries: remodeling, proliferation, senescence, cell apoptosis, and autophagy

The mitogenic response in which mitogen activated protein kinases (MAPKs) and cytokine-mediated signals participate can be reduced by antioxidant enzymes through a direct action on ROS. MAPKs participate in intracellular signal transduction pathways, promoting cell differentiation and survival, arresting growth, apoptosis, and senescence, and thereby generating resistance to radiotherapy and chemotherapy (de la Cruz-Morcillo et al. 2013). The action of ROS as second messenger in inactive receptor transduction continues with the activation of cascade signaling, controlling various cell events such as proliferation, apoptosis, and inflammation. ROS acts on the growth factor stimulus after receptor tyrosine kinases (RTKs) and small GTP proteins –such as Ras and Rac-, and before MAPK members. Ras and Rac proteins appear to be directly related to the production of superoxide anions and therefore to cell transformation. Examples include fibroblasts, whose transformation is largely affected by ROS formation (2003). Depending on the cell system, different members of the MAPK family, including p38 MAPK, ERK1 (kinase 1-regulated extracellular signal), and JNK (c-Jun, the Terminal of N- kinase) have proven to have ROS-sensitive kinase activity. The transduction of the cascade signal induced by ROS, culminates in the phosphorylation and transcriptional activation of c-Jun and c-Fos subunits of the AP-1 active nuclear transcription factor (activating protein 1), which in turn activates genes involved in cell proliferation (Behrend et al. 2003; Valko et al. 2006).

Although the ERK pathway is often activated by mitogenic stimuli, p38 can be stimulated by various stress stimuli. The JNK pathway is involved in responses related to oxidative stress, hence controlling oncogenic expression, and it also mediates in cell death by p53-induced apoptosis (Schramek et al. 2011; Raj et al. 2012). JNK inhibition produces changes in senescence and causes a rapid increase in ROS production in the mitochondria and in the response to DNA damage in breast carcinoma cells (MCF-7). This ROS production is attributed to the suppression of Bcl-2 (B cells of lymphoma 2) phosphorylation, causing DNA damage and stimulating the activation of p53 (Lee et al. 2009). Activation of the JNK signaling pathway involves an anti-tumorigenic response, controlling the oncogene expression. This response is related to the activation of oncogenes that depend on oxidative stress and are controlled by p53 (Schramek et al. 2011).

ROS also intervene in the regulation of the PI3K/AKT (phosphatidyl inositol 3-kinase/protein kinase) pathway, which is related to cell growth, survival, and the activation of transcription factors, e.g., hypoxia inducible factors (HIFs) (Weinberg & Chandel 2009).

ROS play an important role by favoring activation of the autophagy pathway (serine/threonine protein kinase) mTOR in the setting of oxidative stress. Various studies have reported on the role of ROS in the regulation of genes implicated in this process, including Atg4, beclin-1, and p62 (Mathew et al. 2009; Ravikumar et al. 2010; Z-y et al. 2011; Li et al. 2012). Nutritional deficit leads to an ROS increase that eventually affects mTOR *via* the PI3K/AKT pathway, thereby activating autophagy. PI3K is altered in different tumor types, with consequent effects on the autophagic process (Meley et al. 2006; Scherz-Shouval & Elazar 2007; Wong et al. 2010). Oxidative stress in the tumor microenvironment also leads to mitochondrial and aerobic glycolysis dysfunction. It has been observed that cancerous epithelial cells have increased mitochondrial oxidative capacity, showing an elevated enzymatic activity of cytochrome c oxidase (COX) in comparison to normal ductal epithelial cells (Whitaker-Menezes et al. 2011).

The mitochondrial utilization of NO• involves production of the superoxide anion and of hydrogen peroxide, a species that can spread beyond mitochondria and participate in the modulation of cell proliferation, apoptosis, cell transformation, and cancer. A high level of oxidants has been found in many types of cancer cells, and the introduction of chemical and antioxidant substances can inhibit the proliferation of tumor cells, indicating a critical role for ROS as mediator in the loss of growth control (Behrend et al. 2003; Galli et al. 2003; Baty et al. 2005). NO• is one of the most powerful apoptosis regulators, capable of both inducing and blocking this type of programmed cell death (Donovan et al. 2001; Bulotta et al. 2001). The balance between cancer and cell development is represented by levels of cyclin D1 and MAPKs in tumors and tissues in development. The expression of cyclin D1, a protein involved in cell proliferation control and activation of the pro-proliferative protein ERK1/2 or pro-apoptotic protein p38 MAPK in rat liver tumor cells, is subject to dual effects of H_2_O_2_: exposure to high concentrations of H_2_O_2_ increases the expression of p38 and decreases the expression of ERK1 (Chodosh 2002; Galli et al. 2003).

ROS favor the cell senescence observed in the response to constitutively active MAPK proteins. Thus, H_2_O_2_ produces senescence by halting growth in G1, which is accompanied by an increase in the regulation of tumor suppressor p53 and its transcriptional target p21 (Chen et al. 1998; Behrend et al. 2003). Interestingly, the overexpression of p21 induces an increase in ROS levels, favoring cell senescence. Hence, ROS levels act as key mediators in cell transformation, inducing cell cycle arrest, apoptosis, and cell senescence (Behrend et al. 2003; Baty et al. 2005).

The overexpression of the Nox1 gene (analog of NADPH oxidase) in NIH 3T3 fibroblasts of mice induces excess production of ROS and a transformed phenotype. Consequently, PI3-K (phosphoinositide-e-kinase), RAC, and NADPH oxidase are key players in ROS-mediated cell transformation processes. Rac1 and NADPH oxidase, by producing the O_2_^-^•, are also involved in death by apoptosis. This Nox1 overexpression is also observed in different prostate cancer cell lines (PC3, DU145, LNCaP) compared to normal cells, resulting in an increase in ROS (e.g., O_2_^-^• and H_2_O_2_), indicating that the NAD(P)H oxidase (Nox) system is involved in the extra-mitochondrial generation of ROS and plays a key role in the development of the malignant phenotype in prostate cancer cells (Kumar et al. 2008; Luo et al. 2009).

Different members of the Bcl-2 and Bcl-xL family function as antagonists of ROS production in apoptosis, protecting cells from apoptotic induction by exogenous oxidants (Behrend et al. 2003). One example of the capacity of ROS to induce apoptosis is the action of surfactin, a lipopeptide produced by *B.subtilisc* which has antitumor, antimicrobial, and anti-mycoplasma activity that phosphorylates JNK in breast cancer cells (MCF-7). This capacity was demonstrated by using N-acetylcysteine/catalase (antioxidant action) to block ROS, which led to the inhibition of apoptosis in the MCF-7 cells (Cao et al. 2010).

### Free radicals and growth factors

According to recent studies, ROS/RNS participate in the activation of hepatic stellate cells, which are characterized by the production of extracellular matrix and intense proliferation. The interference between parenchymal cells and non-parenchymal cells plays a major role in hepatic damage and fibrogenesis. ROS participate in fibrogenesis by increasing expression of the platelet growth factor (Muriel 2009; Iglesias-de la Cruz et al. 2001). Most hepatocellular carcinomas originate in cirrhotic livers, and a common hepatocarcinogenesis mechanism is chronic inflammation associated with severe oxidative stress. NO• expression can inhibit or promote tumor development, according to the cell setting and concentration. In its pro-tumorigenic role, NO• induces DNA damage and enhances angiogenesis by stimulating the vascular endothelial growth factor (VEGF), contributing to tumor growth and cell invasion. In a mouse model, studies on inflammation-related colon cancer demonstrated that the genetic deletion of NOS2 may lead to a reduction in tumorigenic capacity and that the inhibition of its activity may reduce the tumor load. Conversely, increased NO• levels also have anti-tumorigenic effects, killing malignant cells and enabling the immune system to eliminate cancerous cells (Galli et al. 2003;Vesper et al. 2010). In breast cancer, A case–control study of serum VEGF levels in breast cancer patients reported elevated malondialdehyde (MDA) levels and TAS levels in the cancer patients (Pande et al. 2011). The same authors also demonstrated a positive correlation between oxidative damage, estimated as 8-hydroxy-2-deoxyguanosine (8-OHdG) levels, and the progression of breast cancer (Pande et al. 2012).

ROS production in tumors induces the regulation of nuclear genes associated with higher metastatic potential: MCL-1 (anti-apoptotic, myeloid leukemia cell 1), HIF-1α (hypoxia-inducible factor 1α), and VEGF (Mena et al. 2009; Ishikawa et al. 2008). It has been observed that in highly invasive breast tumor cells, such as MDA-MB-231, the mechanisms that regulate intracellular levels of ROS participate in inhibiting both invasion and migration through a deficiency in OLA1 (negative regulator gene of the antioxidant cell response) (J-w et al. 2009).

### Free radicals and tumor suppressor genes

ROS act directly or indirectly on a wide range of molecules, including protein tyrosine phosphatase (PTP), whose activity is susceptible to the cell redox state and is mainly modified by ROS. PTP has emerged as a type of receptor for ROS signaling, and its activation initiates a signal transduction flow in which MAPKs frequently intervene. At the end point of this pathway, signals are generally connected to the transcriptional activity in the nucleus. In some cases, signals promote cell proliferation by inducing the expression of proto-oncogenes, including c-fos and c-myc (Mori et al. 2004; Luo et al. 2009).

The increase in NO• has pro-tumorigenic or anti-tumorigenic effects according to the status of the p53 tumor suppressor gene. There is a negative feedback loop between NO• and p53 (Ambs et al. 1998): NO• produces stabilization and accumulation of p53, while the activation of p53 suppresses NOS2. Therefore, NO• leads to an increase in the activity of p53, which in turn promotes apoptosis, cell cycle arrest, and senescence in damaged cells. Hence, NO• may have anti-tumorigenic properties. In the absence of p53, there are cells that are not sensitive to NO• -induced apoptosis or cell cycle arrest; however, in other cell types, NO• can induce cell proliferation. A lesser induction of sarcomas and lymphomas was observed in NOS2-deleted mice that lacked p53, which is compatible with the idea that p53 and NO• cooperate to regulate tumor formation. NO• has antitumor activity, inhibiting cell proliferation, promoting differentiation, and reducing the spread of metastases spreading in different types of tumor cells; it also has pro-tumor activity, favoring tumor growth (Forrester et al. 1996; Brennan et al. 2002; Galli et al. 2003; Zhou et al. 2012).

The transcriptional targets of p53 tumor suppressor gene include antioxidant enzymes and RRM2B genes that regulate the codification of the ribonucleotide reductase subunit, preventing DNA dysfunction in the mitochondrion. The ATM gene is a critical mediator in the response to DNA damage and was also found to stabilize mitochondrial DNA by regulating ribonucleotide reductases. These observations reveal the important role of ROS in tumorigenesis and suggest that optimization of the mitochondrial function (e.g. by redox metabolism and maintaining intracellular oxygen homeostasis) may have a protective role against oxidative damage of genomic DNA (Sung et al. 2010; Goh et al. 2011). Sablina et al. (2005) reported that low concentrations of p53 favor the expression of antioxidant genes under conditions of low cell stress, whereas p53 shows oxidant function to favor the expression of genes that promote both an increase in ROS and induction to apoptosis (Sablina et al. 2005).

BRCA1 is involved in ROS production and in the response to oxidative stress, exerting antioxidant activity by inducing the expression of antioxidant enzymes. It is a multifunctional protein involved in numerous cell processes, including cell cycle control, maintenance of genetic stability, DNA damage repair, apoptosis, and the transcription of different genes. Deficiencies in BRCA1 gene in the presence of H_2_O_2_ induce the exportation of phosphorylated protein Smad3 from the nucleus to cell cytoplasm (cytoplasmic factor that binds with other proteins to activate or inhibit the transcription of specific genes); this reduces the Smad3-Smad4 interaction mediated by TGF-b (growth factor responsible for activating Smads by phosphorylation) and slightly decreases the transcriptional activity of both proteins, as evidenced in studies on the response to oxidative stress in breast cancer cell lines (e.g., MCF-7) (Li et al. 2009). In mice, deficiencies in this gene were found to produce an excess of ROS production and increase their sensitivity to oxidative stress (Li et al. 2009; Xiao et al. 2007).

SIRT3, a tumor suppressor localized in the mitochondrion, is required to maintain mitochondrial integrity and metabolism under conditions of oxidative stress. This gene belongs to a family of sirtuins that regulates a wide variety of intracellular processes and is formed by seven NAD +−dependent deacetylase proteins in different cell compartments, including the nucleus (SIRT1, SIRT6, and SIRT7), mitochondrion (SIRT3, SIRT4, and SIRT5), and cytoplasm (SIRT2) (Kim et al. 2010). A deficiency in SIRT3 was reported to increase O_2_^-^• in the mitochondrion, decreasing apoptosis induced by oxidative stress in response to doses of ionizing radiation from 2 to 5 Gy (Kim et al. 2010; Tao et al. 2010).

SMAR1 tumor suppressor gene (cell cycle and apoptotic gene transcription regulator) is repressed in many cases of high histological grade breast cancer and therefore cannot bind to AKR1-4 (antioxidant activity, blocking ROS, and detoxifying), an action mediated by ATM. Hence, it cannot be translocated to the nucleus to exert its function in DNA repair, increasing the antioxidant activity of AKR and favoring tumor progression (Singh et al. 2010).

Both ROS and RNS (RONS) can be increased by oncogene activation. Thus, the activation of RAS signaling leads to a considerable increase in RONS production and contributes to RAS-induced carcinogenesis. RONS can also inhibit tumor formation in certain cell settings, producing inflammation-induced cell senescence in epithelial cells (Schetter et al. 2010).

### Free radicals and metalloproteases

Metalloproteases (MMPs) belong to a family of proteins characterized by a zinc atom in their catalytic site. The members of this family have an important role in cell biology. MMPs can bind to the cell membrane or be secreted into the tumor microenvironment, favoring the disintegration of the extracellular matrix and thereby permitting cells to invade and metastasize (Artacho-Cordón et al. 2012a). This family includes around 24 members (Leonardo & Pennypacker 2009). Nitric oxide activates MMP-9 by nitrosylation of cysteine residues of the pro-peptide, removing the peptides that maintain their catalytic zinc site inactive (Jian Liu & Rosenberg 2005; Rosenberg 2002). Blockade of ROS production by inhibition of the Nox system with a specific cell proliferation inhibitor (e.g., hydroethidine) modulates the activity of the extracellular signaling cascade regulated by ERK1/2 and p38 MAPK (Gurjar et al. 2001). Finally, it arrests the cell cycle in the G2/M phase, decreases MMP-9 activity, and produces a loss in mitochondrial potential, explaining the increased cell death and reduced cell invasiveness (Kumar et al. 2008).

It has been shown that the overexpression of murine manganese superoxide dismutase (mgsod2), which is dependent on the production of H_2_O_2_, increases the activity of transcription factors critical for MMP expression and also improves MMP-1 promoter activity *via* Ras – MAPK. Furthermore, the overexpression of Sod2 increases the mRNA levels of MMPs-2,-3,-7,-10,-9,-11, enhancing the metastatic capacity of fibrosarcoma cells implanted in immunodeficient mice (Nelson et al. 2003).

It has been observed that the overexpression of human manganese superoxide dismutase (MnSOD) in the MCF-7 breast cancer cell line stimulates the activation of MMP-2 and increases the levels of ROS (Zhang et al. 2002). The activity of MMP-2 can be modulated according to intracellular levels of RONS. Radiation produces ROS, including O_2_^-^• and H_2_O_2_ and MnSOD transforms O_2_^-^• into H_2_O_2_, which in turn activates MMP-2. In contrast, NO• hinders the formation of ROS by competition. The antioxidant enzymes that remove H_2_O_2_, such as CAT and GPx, mentioned above, contribute to MMP-2 inactivation and reduce the tumor invasiveness derived from the action of this MMP (Zhang et al. 2002).

The expression of MMP-3 stimulates the production of Rac1b, a hyperactive form of Rac1 that stimulates ROS production and DNA damage, producing chromosomal instability (Colotta et al. 2009).

### Free radicals and adhesion molecules

Various authors have described cases of post-surgical trauma in which a local inflammatory reaction starts with the focalization of polymorphonuclear (PMN) cells. These activated cells produce ROS that improve the adhesion of tumor cells to the mesothelium, an essential step in tumor progression. A study of the interaction between tumor and endothelial cells in colon and pancreas carcinoma found that levels of adhesion molecules were higher in the presence of the O_2_^-^• (e.g., E-selectin, ICAM-1, and VCAM-1), increasing the adhesion percentage (Ten Kate et al. 2006).

E-cadherin is a critical mediator in cell adhesion, and its alteration is associated with the dissemination of cancer cells and formation of metastasis (Thiery 2002; Wang & Shang 2013). Activation of the Src gene (tyrosine kinase that regulates intracellular signals in cell proliferation) takes places by s-nitrosylation through the action of NO•. β-estradiol-stimulated activation decreases the expression of E-cadherin in MCF-7 breast cancer cell (Rahman et al. 2010). *Mori* et al. observed that E-cadherins change their localization after daily treatment with H_2_O_2_ for 4 days, with signals dispersed throughout cytoplasm as small dots, suggesting a vesicular localization, with residues at cell margins (Mori et al. 2004; Kheradmand et al. 1998).

Tumor cells may present a situation of epithelium-mesenchymal transition (EMT), by which the transformed cells separate from the basal lamina and reorganize their cytoskeleton, favoring their motility and migration through the surrounding tissue (Polyak & Weinberg 2009). Subsequently, the extracellular matrix (ECM) around the primary tumor must be remodeled in order to permit tumor cells enter the bloodstream. Integrins have an important role in the ECM as support and in cell binding, and they also participate in cell signaling and therefore the regulation of various processes, including cell proliferation, survival, and migration. There are different signaling pathways for integrins in breast cancer such as TGF-β, PKC, MAPKs, AKT, NF-κB, and PI3K. Integrin effectors from the Rho-GTPase family, including RhoA, Rac 1, and Cdc42, are involved in the stimulation of cell adhesion and consequently induce ROS production (Vera-Ramirez et al. 2011). In this context, the induction of EMT by ROS modifies the cells of the mammary epithelium and produces Rac-1 overexpression, thereby favoring their invasive capacity (Vera-Ramirez et al. 2011; Mori et al. 2004).

### Free radicals and extracellular matrix

The ECM plays an important role in cell differentiation and apoptosis. Collagen is the main component of the ECM and inhibits the signal of the estrogen receptor (ESR) generator of the hydroxyl radical via Fe^2+^ by the Fenton reaction. Free radicals are important in collagen synthesis and may increase the gene expression of human mesangial cells in the ECM. The hydroxyl radical induces the apoptotic pathway in human tumor cells. Some studies have suggested that collagen blocks the generation of the radical to protect damaged fibroblasts; therefore, collagen remodeling is influenced by free radicals (He et al. 2002; Sethy-Coraci et al. 2005). It has been observed that glucose deprivation may induce ROS production during tumorigenesis, producing a selection of alterations that allow cells to move away from the environment with excess oxidative damage. Antioxidants facilitate the survival of these cells and enhance the formation of cell colonies that are not bound to the ECM. According to these findings, antioxidants promote the survival of cells lacking anchorage to the ECM, which suggests that they may have a dual function in regard to the tumorigenesis process (Schafer et al. 2009).

### Free radicals, inflammation, and cancer

An inflammatory stimulus leads to the recruitment and activation of various immunological cells, including macrophages, neutrophils, and dendritic cells, which facilitate the release and accumulation of RONS. ROS, such as the O_2_^-^• and H_2_O_2_, are released by leucocytes and other phagocytic cells accumulated in infection sites and wounds. These radicals play an important role in the microbiocidal activity of the innate immune response (Grivennikov et al. 2010). Correct RONS regulation is vital for generating an effective immune response and reducing tissue damage. Activated neutrophils use superoxide radicals as a defense mechanism against bacteria, and this excess release of oxidants is induced by growth factors. ROS production in non-phagocytic cells takes place through activation of the RTK growth receptor (receptor of tyrosine kinase), including activation by different factors, such as platelet growth factor, basic fibroblast growth factor, and epidermal growth factor. Cytokines, tumor necrosis factor, interferon-γ, and interleukins are also ROS-inducers (Coussens & Werb 2002; Behrend et al. 2003; Purdom & Chen 2005). Furthermore, inflammation in breast cancer is regulated by c-myb, which is modulated by the MAPK pathway, and ROS participate in this signaling pathway, favoring the proliferation and progression of the cancer (Bhattarai et al. 2011). Research is currently under way on the role of obesity-related oxidative stress in chronic inflammation during the onset and progression of breast cancer (Crujeiras et al. 2013).

The NOS2 enzyme is capable of producing very high levels of NO• in response to inflammatory stimuli. It may also S-nitrosylate COX-2 and increase its proinflammatory activity. NOS2 can induce various factors, including inflammatory cytokines and NF-κβ. NOS2 induction in phagocytic cells, such as monocytes, macrophages, and neutrophils, leads to the overexpression of NO•, a key mediator in the immune-inflammatory response. The cell response is determined by the NO• levels in both tumor cells and the tumor microenvironment (Schetter et al. 2010).

The relationship between inflammation and carcinogenesis is increasingly well documented, with numerous reports of cancer originating at sites of previous chronic inflammation (Grivennikov et al. 2010). Studies have been published on changes in morphology and in gene expression of mouse mammary epithelial cells (NMuMG) after prolonged exposure to H_2_O_2_, which simulates chronic inflammation. Under these oxidation conditions, a phenotypic cell conversion with striking similarities to malignant transformation was observed, including a fibroblastic morphology with intercellular spaces, implying a decrease in intercellular connections (Mori et al. 2004).

RONS and inflammatory cytokines (TNF and IL-1β) induce the expression of HIF-1α in cancerous cells by displacing and negatively regulating the c-Myc of MSH2/MSH6 promoter, members of the MMR family (mismatch repair), which are repairers of insertions and deletions in bases. Hydrogen peroxide inactivates MMRs, damaging the enzymes at protein level. NO• induces the positive regulation of DNA methyltransferase, favoring methylation and producing the loss of expression and silencing of the hMLH1 gene, a member of the MMR family. Both NO• and IL-6 enhance DNA methyltransferase activity (Colotta et al. 2009; Artacho-Cordón et al. 2012b).

NO• induces the hyperphosphorylation of the retinoblastoma protein, releasing the E2F1 factor, which is negatively regulated by Mad2 and is overexpressed in various tumor types, favoring chromosomal instability (Colotta et al. 2009).

**Figure 2:**
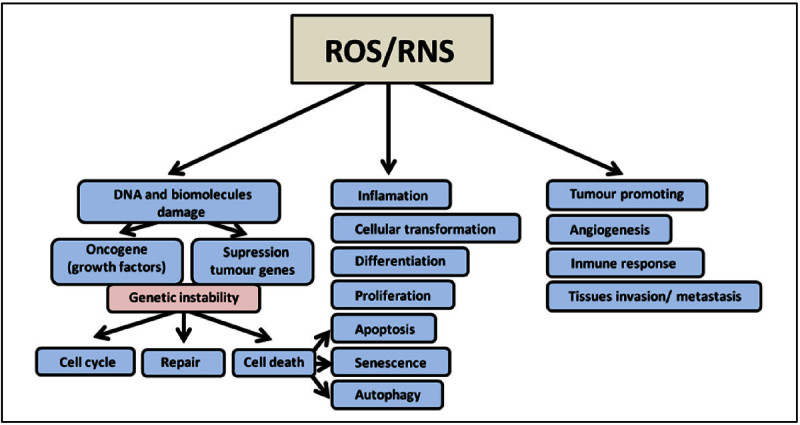
**ROS/RNS role in the carcinogenesis process.** Oxygen and nitrogen reactive species produce damage in the DNA and other biomolecules and play a major role in genetic instability, affecting progression through the cell cycle, cell repair, and the type of cell death (apoptosis, senescence, or autophagy). Free radicals are also important in cell transformation, differentiation, and cell proliferation processes and may be useful for evaluating the tissue inflammatory response. Finally, with respect to carcinogenesis, these radicals have been implicated in tumor progression, angiogenesis, the immune response, and the invasive and metastatic potential of tumor cells.

The •OH produced during inflammation are very harmful and have been implicated in base modifications, including the formation of thymine, thymidine glycol, 8-OHdG and 5-hydroxymethyluracil. 8-OHdG is a modified guanine that induces a point mutation in the daughter DNA strand and is widely used as an indicator of DNA damage (Gl et al. 2011; Kumar et al. 2012). This mutation is one of the most frequent in different cancers (Wood et al. 2007). Figure 2 represents the role of ROS/RNS in the carcinogenesis process as previously described.

### Free radicals, ionizing and non-ionizing radiation

Ionizing radiation falls directly on DNA, generating charged particles or electrons that carry the kinetic energy provided by photons (X rays, γ rays), producing breaks in phosphodiester bonds. This represents around 30% of DNA damage (Jeggo & Lavin 2009; Kempner 2011). The remaining damage derives from the action of free radicals. The •OH, which has high biological relevance, is generated by the interaction of ionizing radiation (e.g., X- or gamma rays) with the water molecule in a process known as water “radiolysis”. The deposition of energy from radiation also generates hydrogen atoms and hydrated electrons and other molecular products. These include molecular hydrogen, hydrogen peroxide, and peroxynitrite, compounds that generate DNA-damage like 8-hydroxyguanine (8-OH-Gua), 8-OH-dG, 8-oxoguanine and consequently single- and double-strand DNA breaks (Gl et al. 2011).

As already noted, free radicals are important factors in carcinogenesis (Anastassopoulou & Theophanides 2002; Marnett 2000). In the human body, the •OH has an extraordinarily short life due to collisions with the different and abundant molecules in the biological environment. Scientists have concluded that radiotherapy generates the •OH, which is the free radical most associated with cell death. For its part, the NO• appears to act as radiosensitizer under conditions of hypoxia, mimicking the effects of oxygen relating on radiation-induced DNA damage (De Ridder et al. 2008;Oronsky et al. 2012;Folkes & O'Neill 2013). The main late complication after radiotherapy in breast cancer is the fibrosis that results from radiation-induced inflammatory responses (Paquette et al. 2007).

Studies on the levels of redox protein and ionizing radiation in breast cancer patients have shown the high cytoplasmic expression of glutathione-s-transferase and low cytoplasmic and nuclear expression of glutathione-peroxidase 3 are correlated with an increase in the risk of local recurrence. Regulation of the redox system in these patients may contribute to protect them against the oxidative stress induced by ionizing radiation (Woolston et al. 2011).

*Murley* and coworkers observed elevated SOD2 activity when the RKO36 cell line was exposed to a 2Gy X-ray dose and reported a higher resistance to radiation in exposed cells, known as the effect of a response adapted to low radiation doses. According to these authors, SOD2 plays an important role in this response impairment by inducing the superoxide anion, which triggers a ROS cascade that produces damage in the cell nucleus and mitochondria (Murley et al. 2011). It was found that SOD activity is higher in malignant *versus* benign breast tumors, which appears to indicate that excess ROS may predict carcinogenesis (Hasan et al. 2012).

In the case of non-ionizing radiations, exposure to radiofrequency electromagnetic waves (RF-EMW) for a short time period may increase the presence of NADH oxidase enzymes in the cell membrane, augmenting ROS production (Friedman et al. 2007). ROS activate MMPs, which release the epidermal growth factor and activate ERK. Chronic exposure to RF-EMW induces kinase alterations and activates p38 MAPK, which in turn induces phosphorylation through the thermal shock of proteins inhibiting the apoptosis pathway (Leszczynski et al. 2002). Hence, radiation from mobile telephony may accumulate damage in cell DNA, producing uncontrolled cell proliferation. Chronic exposure to RF-EMW decreases the activity of different catalase enzymes, such as SOD and GPx, reducing the antioxidant capacity of cells (Desai et al. 2009). It has also been observed that exposure to extremely low frequency electromagnetic fields (ELF-EMF) induces DNA damage in different tumor cell lines. The effect of ELF-EMF on cell proliferation and DNA damage in studied tumor cell lines is inhibited by pretreatment with antioxidants, demonstrating the role of the redox state in cells (Wolf et al. 2005).

The well-documented relationship of free radicals with carcinogenesis makes these species promising candidates as therapeutic targets in cancer, among other diseases. Considerable research efforts are required to fully elucidate their influence on different tumor cell lines and to understand the mechanisms by which they can respond against damage induced by ionizing radiation, widely used in cancer therapy.

## Authors’ original submitted files for images

Below are the links to the authors’ original submitted files for images. Authors’ original file for figure 1 Authors’ original file for figure 2

## References

[CR1] Ambs S, Ogunfusika MO, Merriam WG, Bennett WP, Billiar TR, Harris CC (1998). Up-regulation of inducible nitric oxide synthase expression in cancer-prone p53 knockout mice. Proc Natl Acad Sci.

[CR2] Anastassopoulou J, Theophanides T (2002). Magnesium-DNA interactions and the possible relation of magnesium to carcinogenesis. Irradiation and free radicals. Crit Rev Oncol Hematol.

[CR3] Artacho-Cordón F, Ríos-Arrabal S, Lara PC, Artacho-Cordón A, Calvente I, Núñez MI (2012). Matrix metalloproteinases: Potential therapy to prevent the development of second malignancies after breast radiotherapy. Surgical oncology.

[CR4] Artacho-Cordón A, Artacho-Cordón F, Ríos-Arrabal S, Calvente I, Núñez MI (2012). Tumor microenvironment and breast cancer progression: A complex scenario. Cancer Biol Ther.

[CR5] Badjatia N, Satyam A, Singh P, Seth A, Sharma A (2010). Altered antioxidant status and lipid peroxidation in Indian patients with urothelial bladder carcinoma. Urol Oncol.

[CR6] Baty JW, Hampton MB, Winterbourn CC (2005). Proteomic detection of hydrogen peroxide-sensitive thiol proteins in Jurkat cells. Biochem J.

[CR7] Behrend L, Henderson G, Zwacka R (2003). Reactive oxygen species in oncogenic transformation. Biochem Soc Trans.

[CR8] Bhattarai G, Lee YH, Lee NH, Yun JS, Hwang PH, Yi HK (2011). c-myb mediates inflammatory reaction against oxidative stress in human breast cancer cell line, MCF-7. Cell Biochem Funct.

[CR9] Brennan PA, Umar T, Smith GI, McCauley P, Peters WJ, Langdon JD (2002). Expression of type 2 nitric oxide synthase and p53 in Warthin's tumour of the parotid. J Oral Pathol Med.

[CR10] Brigelius-Flohé R, Kipp A (2009). Glutathione peroxidases in different stages of carcinogenesis. Biochimica et Biophysica Acta (BBA) - General Subjects.

[CR11] Bulotta S, Barsacchi R, Rotiroti D, Borgese N, Clementi E (2001). Activation of the endothelial nitric-oxide synthase by tumor necrosis factor-alpha a novel feedback mechanism regulating cell death. J Biol Chem.

[CR12] Cadenas E, Sies H (1998). The lag phase. Free radical Res.

[CR13] Cao X-h, Wang A-h, Wang C-l, Mao D-z, Lu M-f, Cui Y-q, Jiao R-z (2010). Surfactin induces apoptosis in human breast cancer MCF-7 cells through a ROS/JNK-mediated mitochondrial/caspase pathway. Chemico-biological interactions.

[CR14] Chen QM, Bartholomew JC, Campisi J, Acosta M, Reagan JD, Ames B (1998). Molecular analysis of H2O2-induced senescent-like growth arrest in normal human fibroblasts: p53 and Rb control G1 arrest but not cell replication. Biochem J.

[CR15] Chodosh LA (2002). The reciprocal dance between cancer and development. N Engl J Med.

[CR16] Colotta F, Allavena P, Sica A, Garlanda C, Mantovani A (2009). Cancer-related inflammation, the seventh hallmark of cancer: links to genetic instability. Carcinogenesis.

[CR17] Coussens LM, Werb Z (2002). Inflammation and cancer. Nature.

[CR18] Crujeiras AB, Díaz-Lagares A, Carreira MC, Amil M, Casanueva FF (2013). Oxidative stress associated to dysfunctional adipose tissue: a potential link between obesity, type 2 diabetes mellitus and breast cancer. Free radical research.

[CR19] Cuzzocrea S, Riley DP, Caputi AP, Salvemini D (2001). Antioxidant therapy: a new pharmacological approach in shock, inflammation, and ischemia/reperfusion injury. Pharmacol Rev.

[CR20] Davies K, Delsignore M (1987). Protein damage and degradation by oxygen radicals. III. Modification of secondary and tertiary structure. J Biol Chem.

[CR21] de la Cruz-Morcillo MA, García-Cano J, Arias-González L, García-Gil E, Artacho-Cordón F, Ríos-Arrabal S, Valero ML, Cimas FJ, Serrano-Oviedo L, Villas MV (2013). Abrogation of the p38 MAPK α signaling pathway does not promote radioresistance but its activity is required for 5-Fluorouracil-associated radiosensitivity. Cancer letters.

[CR22] De Ridder M, Verellen D, Verovski V, Storme G (2008). Hypoxic tumor cell radiosensitization through nitric oxide. Nitric oxide.

[CR23] Delwar ZM, Vita MF, Åk S, Cruz M, Yakisich JS (2011). In vitro inhibition of topoisomerase IIα by reduced glutathione. Acta biochimica Polonica.

[CR24] Desai NR, Kesari KK, Agarwal A (2009). Pathophysiology of cell phone radiation: oxidative stress and carcinogenesis with focus on male reproductive system. Reprod Biol Endocrinol.

[CR25] Donovan M, Carmody RÌJ, Cotter TG (2001). Light-induced photoreceptor apoptosis in vivo requires neuronal nitric-oxide synthase and guanylate cyclase activity and is caspase-3-independent. J Biol Chem.

[CR26] Dudzinski DM, Michel T (2007). Life history of eNOS: partners and pathways. Cardiovasc Res.

[CR27] Ferrari CKB, Franca EL, Honorio-Franca AC (2009). Nitric oxide, health and disease. J Appl Biomed.

[CR28] Folkes LK, O'Neill P (2013). Modification of DNA damage mechanisms by nitric oxide during ionizing radiation. Free Radical Biol Med.

[CR29] Forrester K, Ambs S, Lupold SE, Kapust RB, Spillare EA, Weinberg WC, Felley-Bosco E, Wang XW, Geller DA, Tzeng E (1996). Nitric oxide-induced p53 accumulation and regulation of inducible nitric oxide synthase expression by wild-type p53. Proc Natl Acad Sci.

[CR30] Friedman J, Kraus S, Hauptman Y, Schiff Y, Seger R (2007). Mechanism of short-term ERK activation by electromagnetic fields at mobile phone frequencies. Biochem J.

[CR31] Fuchs-Tarlovsky V (2013). Role of antioxidants in cancer therapy. Nutrition (Burbank, Los Angeles County, Calif).

[CR32] Galli S, Labato MI, de Kier JoffÃ© EB, Carreras MC, Poderoso JJ (2003). Decreased mitochondrial nitric oxide synthase activity and hydrogen peroxide relate persistent tumoral proliferation to embryonic behavior. Cancer research.

[CR33] Gl K, Keles D, Canda AE, Terzi C, Reddy PT, Jaruga P, Dizdaroglu M, Gln O (2011). Evidence for upregulated repair of oxidatively induced DNA damage in human colorectal cancer. DNA repair.

[CR34] Goh J, Enns L, Fatemie S, Hopkins H, Morton J, Pettan-Brewer C, Ladiges W (2011). Mitochondrial targeted catalase suppresses invasive breast cancer in mice. BMC cancer.

[CR35] Griendling KK, Sorescu D, LassÃ¨gue B, Ushio-Fukai M (2000). Modulation of protein kinase activity and gene expression by reactive oxygen species and their role in vascular physiology and pathophysiology. Arteriosclerosis, thrombosis, and vascular biology.

[CR36] Griffith OW (1999). Biologic and pharmacologic regulation of mammalian glutathione synthesis. Free Radical Biol Med.

[CR37] Grivennikov SI, Greten FR, Karin M (2010). Immunity, inflammation, and cancer. Cell.

[CR38] Gurjar MV, Deleon J, Sharma RV, Bhalla RC (2001). Role of reactive oxygen species in IL-1b-stimulated sustained ERK activation and MMP-9 induction. Am J Physiol Heart Circ Physiol.

[CR39] Halliwell B (1999). Oxygen and nitrogen are pro-carcinogens. Damage to DNA by reactive oxygen, chlorine and nitrogen species: measurement, mechanism and the effects of nutrition. Mutat Res–Genetic Toxicol Environ Mutagen.

[CR40] Halliwell B, Gutteridge J (2007). Free radicals in biology and medicine.

[CR41] Hasan HR, Mathkor TH, Al-Habal MH (2012). Superoxide Dismutase Isoenzyme Activities in Plasma and Tissues of Iraqi Patients with Breast Cancer. Asian Pac J Cancer Prev.

[CR42] He Y, Chen J, Ren J, Wang G, Cai G (2002). Type I collagen inhibits hydroxyl radical-induced apoptosis. J Biochem.

[CR43] Iglesias-de la Cruz MC, Ruiz-Torres P, Alcami J, Diez-Marques L, Ortega-Velazquez R, Chen S, Rodriguez-Puyol M, Ziyadeh FN, Rodríguez-Puyol D (2001). Hydrogen peroxide increases extracellular matrix mRNA through TGF-b in human mesangial cells. Kidney international.

[CR44] Iolascon A, De Falco L, Beaumont C (2009). Molecular basis of inherited microcytic anemia due to defects in iron acquisition or heme synthesis. haematologica.

[CR45] Ishikawa K, Takenaga K, Akimoto M, Koshikawa N, Yamaguchi A, Imanishi H, Nakada K, Honma Y, Hayashi J-I (2008). ROS-generating mitochondrial DNA mutations can regulate tumor cell metastasis. Sci Signal.

[CR46] Jeggo P, Lavin MF (2009). Cellular radiosensitivity: how much better do we understand it?. Int J Radiat Biol.

[CR47] Jian Liu K, Rosenberg GA (2005). Matrix metalloproteinases and free radicals in cerebral ischemia. Free Radical Biol Med.

[CR48] Jomova K, Valko M (2011). Importance of iron chelation in free radical-induced oxidative stress and human disease. Curr Pharm Des.

[CR49] Jones DP, Carlson JL, Mody VC, Cai J, Lynn MJ, Sternberg P (2000). Redox state of glutathione in human plasma. Free Radical Biol Med.

[CR50] J-w Z, Rubio V, Zheng S, Z-z S (2009). Knockdown of OLA1, a regulator of oxidative stress response, inhibits motility and invasion of breast cancer cells. Journal of Zhejiang University SCIENCE B.

[CR51] Kempner ES (2011). Direct effects of ionizing radiation on macromolecules. J Polym Sci B.

[CR52] Kheradmand F, Werner E, Tremble P, Symons M, Werb Z (1998). Role of Rac1 and oxygen radicals in collagenase-1 expression induced by cell shape change. Science.

[CR53] Kim J, Gherasim C, Banerjee R (2008). Decyanation of vitamin B12 by a trafficking chaperone. Proc Natl Acad Sci.

[CR54] Kim H-S, Patel K, Muldoon-Jacobs K, Bisht KS, Aykin-Burns N, Pennington JD, van der Meer R, Nguyen P, Savage J, Owens KM (2010). SIRT3 is a mitochondria-localized tumor suppressor required for maintenance of mitochondrial integrity and metabolism during stress. Cancer cell.

[CR55] Klaunig JE, Kamendulis LM (2004). The role of oxidative stress in carcinogenesis. Annu Rev Pharmacol Toxicol.

[CR56] Klaunig JE, Kamendulis LM, Hocevar BA (2010). Oxidative stress and oxidative damage in carcinogenesis. Toxicologic pathology.

[CR57] Kumar B, Koul S, Khandrika L, Meacham RB, Koul HK (2008). Oxidative stress is inherent in prostate cancer cells and is required for aggressive phenotype. Cancer research.

[CR58] Kumar A, Pant M, Singh H, Khandelwal S (2012). Assessment of the redox profile and oxidative DNA damage (8-OHdG) in squamous cell carcinoma of head and neck. J Cancer Res Ther.

[CR59] Lee JJ, Lee JH, Ko YG, Hong SI, Lee JS (2009). Prevention of premature senescence requires JNK regulation of Bcl-2 and reactive oxygen species. Oncogene.

[CR60] Leonardo CC, Pennypacker KR (2009). Neuroinflammation and MMPs: potential therapeutic targets in neonatal hypoxic-ischemic injury. J Neuroinflammation.

[CR61] Leszczynski D, Joenväärä S, Reivinen J, Kuokka R (2002). Non-thermal activation of the hsp27/p38MAPK stress pathway by mobile phone radiation in human endothelial cells: molecular mechanism for cancer- and blood–brain barrier-related effects. Differentiation.

[CR62] Li H, Sekine M, Seng S, Avraham S, Avraham HK (2009). BRCA1 Interacts with Smad3 and Regulates Smad3-Mediated TGF-b Signaling during Oxidative Stress Responses. PloS one.

[CR63] Li L, Ishdorj G, Gibson SB (2012). Reactive oxygen species regulation of autophagy in cancer: Implications for cancer treatment. Free Radical Biol Med.

[CR64] Luo J, Solimini NL, Elledge SJ (2009). Principles of cancer therapy: oncogene and non-oncogene addiction. Cell.

[CR65] Mahelkova G, Korynta J, Moravova A, Novotna J, Vytasek R, Wilhelm J (2008). Changes of extracellular matrix of rat cornea after exposure to hypoxia. Physiol Res.

[CR66] Marnett LJ (2000). Oxyradicals and DNA damage. Carcinogenesis.

[CR67] Martínez Sarrasague M, Barrado DA, Zubillaga M, Hager A, De Paoli T, Boccio J (2006). Conceptos actuales del metabolismo del glutatión Utilización de los isótopos estables para la evaluación de su homeostasis. Acta bioquímica clínica latinoamericana.

[CR68] Mathew R, Karp CM, Beaudoin B, Vuong N, Chen G, Chen H-Y, Bray K, Reddy A, Bhanot G, Gelinas C (2009). Autophagy suppresses tumorigenesis through elimination of p62. Cell.

[CR69] Meley D, Bauvy C, Houben-Weerts JH, Dubbelhuis PF, Helmond MT, Codogno P, Meijer AJ (2006). AMP-activated protein kinase and the regulation of autophagic proteolysis. J Biol Chem.

[CR70] Mena S, Ortega A, Estrela JM (2009). Oxidative stress in environmental-induced carcinogenesis. Mutat Res–Genetic Toxicol Environ Mutagen.

[CR71] Mori K, Shibanuma M, Nose K (2004). Invasive potential induced under long-term oxidative stress in mammary epithelial cells. Cancer research.

[CR72] Muriel P (2009). Role of free radicals in liver diseases. Hepatology international.

[CR73] Murley JS, Kataoka Y, Miller RC, Li JJ, Woloschak G, Grdina DJ (2011). SOD2-mediated effects induced by WR1065 and low-dose ionizing radiation on micronucleus formation in RKO human colon carcinoma cells. Radiation research.

[CR74] Nathan C (1992). Nitric oxide as a secretory product of mammalian cells. FASEB J.

[CR75] Nelson KK, Ranganathan AC, Mansouri J, Rodriguez AM, Providence KM, Rutter JL, Pumiglia K, Bennett JA, Melendez JA (2003). Elevated Sod2 Activity Augments Matrix Metalloproteinase Expression Evidence for the Involvement of Endogenous Hydrogen Peroxide in Regulating Metastasis. Clin Cancer Res.

[CR76] Okezie I, Harparkash K, Haliwell B (1991). Oxygen free radicals and human diseases. J Roy Soc Health.

[CR77] Oronsky BT, Knox SJ, Scicinski JJ (2012). Is nitric oxide (NO) the last word in radiosensitization? A review. Translational Oncology.

[CR78] Pande D, Negi R, Khanna S, Khanna R, Khanna HD (2011). Vascular endothelial growth factor levels in relation to oxidative damage and antioxidant status in patients with breast cancer. Journal of breast cancer.

[CR79] Pande D, Negi R, Karki K, Khanna S, Khanna RS, Khanna HD (2012). Oxidative damage markers as possible discriminatory biomarkers in breast carcinoma. Transl Res.

[CR80] Pani G, Galeotti T, Chiarugi P (2010). Metastasis: cancer cell's escape from oxidative stress. Cancer Metastasis Rev.

[CR81] Panis C, Herrera AC, Victorino VJ, Campos FC, Freitas LF, De Rossi T, Colado Simão AN, Cecchini AL, Cecchini R (2012). Oxidative stress and hematological profiles of advanced breast cancer patients subjected to paclitaxel or doxorubicin chemotherapy. Breast Cancer Res Treat.

[CR82] Paquette B, Baptiste C, Therriault H, Arguin G, Plouffe B, Lemay R (2007). In vitro irradiation of basement membrane enhances the invasiveness of breast cancer cells. Brit J Cancer.

[CR83] Pervin S, Tran L, Urman R, Braga M, Parveen M, Li S, Chaudhuri G, Singh R (2013). Oxidative stress specifically downregulates survivin to promote breast tumour formation. Brit J Cancer.

[CR84] Polidori MC, Stahl W, Eichler O, Niestroj I, Sies H (2001). Profiles of antioxidants in human plasma. Free Radical Biol Med.

[CR85] Polyak K, Weinberg RA (2009). Transitions between epithelial and mesenchymal states: acquisition of malignant and stem cell traits. Nat Rev Cancer.

[CR86] Pourahmad J, O'Brien PJ, Jokar F, Daraei B (2003). Carcinogenic metal induced sites of reactive oxygen species formation in hepatocytes. Toxicol In Vitro.

[CR87] Prousek J (2007). Fenton chemistry in biology and medicine. Pure Appl Chem.

[CR88] Purdom S, Chen QM (2005). Epidermal growth factor receptor-dependent and-independent pathways in hydrogen peroxide-induced mitogen-activated protein kinase activation in cardiomyocytes and heart fibroblasts. J Pharmacol Exp Ther.

[CR89] Quievryn G, Messer J, Zhitkovich A (2002). Carcinogenic chromium (VI) induces cross-linking of vitamin C to DNA in vitro and in human lung A549 cells. Biochemistry.

[CR90] Rahman MA, Senga T, Ito S, Hyodo T, Hasegawa H, Hamaguchi M (2010). S-nitrosylation at cysteine 498 of c-Src tyrosine kinase regulates nitric oxide-mediated cell invasion. J Biol Chem.

[CR91] Raj L, Ide T, Gurkar AU, Foley M, Schenone M, Li X, Tolliday NJ, Golub TR, Carr SA, Shamji AF (2012). Selective killing of cancer cells by a small molecule targeting the stress response to ROS. Nature.

[CR92] Ravikumar B, Sarkar S, Davies JE, Futter M, Garcia-Arencibia M, Green-Thompson ZW, Jimenez-Sanchez M, Korolchuk VI, Lichtenberg M, Luo S (2010). Regulation of mammalian autophagy in physiology and pathophysiology. Physiol Rev.

[CR93] Reynolds M, Stoddard L, Bespalov I, Zhitkovich A (2007). Ascorbate acts as a highly potent inducer of chromate mutagenesis and clastogenesis: linkage to DNA breaks in G2 phase by mismatch repair. Nucleic Acids Res.

[CR94] Ridnour LA, Thomas DD, Mancardi D, Espey MG, Miranda KM, Paolocci N, Feelisch M, Fukuto J, Wink DA (2004). The chemistry of nitrosative stress induced by nitric oxide and reactive nitrogen oxide species. Putting perspective on stressful biological situations. Biol Chem.

[CR95] Roberts RA, Laskin DL, Smith CV, Robertson FM, Allen EM, Doorn JA, Slikker W (2009). Nitrative and oxidative stress in toxicology and disease. Toxicological sciences.

[CR96] Rosenberg GA (2002). Matrix metalloproteinases in neuroinflammation. Glia.

[CR97] Sablina AA, Budanov AV, Ilyinskaya GV, Agapova LS, Kravchenko JE, Chumakov PM (2005). The antioxidant function of the p53 tumor suppressor. Nature medicine.

[CR98] Schafer FQ, Buettner GR (2001). Redox environment of the cell as viewed through the redox state of the glutathione disulfide/glutathione couple. Free Radical Biol Med.

[CR99] Schafer ZT, Grassian AR, Song L, Jiang Z, Gerhart-Hines Z, Irie HY, Gao S, Puigserver P, Brugge JS (2009). Antioxidant and oncogene rescue of metabolic defects caused by loss of matrix attachment. Nature.

[CR100] Scherz-Shouval R, Elazar Z (2007). ROS, mitochondria and the regulation of autophagy. Trends in cell Biol.

[CR101] Schetter AJ, Heegaard NH, Harris CC (2010). Inflammation and cancer: interweaving microRNA, free radical, cytokine and p53 pathways. Carcinogenesis.

[CR102] Schramek D, Kotsinas A, Meixner A, Wada T, Elling U, Pospisilik JA, Neely GG, Zwick R-H, Sigl V, Forni G (2011). The stress kinase MKK7 couples oncogenic stress to p53 stability and tumor suppression. Nature genetics.

[CR103] Sethy-Coraci I, Crock LW, Silverstein SC (2005). PAF-receptor antagonists, lovastatin, and the PTK inhibitor genistein inhibit H2O2 secretion by macrophages cultured on oxidized-LDL matrices. Journal of leukocyte biology.

[CR104] Shen K, Ji L, Chen Y, Yu Q, Wang Z (2011). Influence of glutathione levels and activity of glutathione-related enzymes in the brains of tumor-bearing mice. Bioscience trends.

[CR105] Singh S, Gupta AK (2011). Nitric oxide: role in tumour biology and iNOS/NO-based anticancer therapies. Cancer chemotherapy and pharmacology.

[CR106] Singh S, Sreenath K, Pavithra L, Roy S, Chattopadhyay S (2010). SMAR1 regulates free radical stress through modulation of AKR1a4 enzyme activity. Int J Biochem Cell Biol.

[CR107] Skrzydlewska E, Sulkowski S, Koda M, Zalewski B, Kanczuga-Koda L, Sulkowska M (2005). Lipid peroxidation and antioxidant status in colorectal cancer. World J Gastroenterol.

[CR108] Speisky H, Gómez M, Burgos-Bravo F, López-Alarcón C, Jullian C, Olea-Azar C, Aliaga ME (2009). Generation of superoxide radicals by copper-glutathione complexes: Redox-consequences associated with their interaction with reduced glutathione. Bioorg Med Chem.

[CR109] Sung HJ, Ma W, P-y W, Hynes J, O'Riordan TC, Combs CA, McCoy JP, Bunz F, Kang J-G, Hwang PM (2010). Mitochondrial respiration protects against oxygen-associated DNA damage. Nat Commun.

[CR110] Tao R, Coleman MC, Pennington JD, Ozden O, Park S-H, Jiang H, Kim H-S, Flynn CR, Hill S, Hayes McDonald W (2010). Sirt3-mediated deacetylation of evolutionarily conserved lysine 122 regulates MnSOD activity in response to stress. Molecular cell.

[CR111] Teixeira V, Valente H, Casal S, Marques F, Moreira P (2013). Blood antioxidant and oxidative stress biomarkers acute responses to a 1000-m kayak sprint in elite male kayakers. J Sports Med physical fitness.

[CR112] Ten Kate M, van der Wal J, Sluiter W, Hofland L, Jeekel J, Sonneveld P, van Eijck C (2006). The role of superoxide anions in the development of distant tumour recurrence. Brit J Cancer.

[CR113] Thiery JP (2002). Epithelial-mesenchymal transitions in tumour progression. Nat Rev Cancer.

[CR114] Turi JL, Jaspers I, Dailey LA, Madden MC, Brighton LE, Carter JD, Nozik-Grayck E, Piantadosi CA, Ghio AJ (2002). Oxidative stress activates anion exchange protein 2 and AP-1 in airway epithelial cells. Am J Physiol Lung Cell Mol Physiol.

[CR115] Valko M, Rhodes C, Moncol J, Izakovic M, Mazur M (2006). Free radicals, metals and antioxidants in oxidative stress-induced cancer. Chemico-biological interactions.

[CR116] Valko M, Leibfritz D, Moncol J, Cronin MT, Mazur M, Telser J (2007). Free radicals and antioxidants in normal physiological functions and human disease. Int J Biochem Cell Biol.

[CR117] Vera-Ramirez L, Sanchez-Rovira P, Ramirez-Tortosa MC, Ramirez-Tortosa CL, Granados-Principal S, Lorente JA, Quiles JL (2011). Free radicals in breast carcinogenesis, breast cancer progression and cancer stem cells. Biological bases to develop oxidative-based therapies. Crit Rev Oncol Hematol.

[CR118] Vesper BJ, Elseth KM, Tarjan G, Haines GK, Radosevich JA (2010). Long-term adaptation of breast tumor cell lines to high concentrations of nitric oxide. Tumor Biology.

[CR119] Wang Y, Shang Y (2013). Epigenetic control of epithelial-to-mesenchymal transition and cancer metastasis. Exp Cell Res.

[CR120] Weinberg F, Chandel NS (2009). Reactive oxygen species-dependent signaling regulates cancer. Cellular and molecular life sciences.

[CR121] Whitaker-Menezes D, Martinez-Outschoorn UE, Flomenberg N, Birbe RC, Witkiewicz AK, Howell A, Pavlides S, Tsirigos A, Ertel A, Pestell RG (2011). Hyperactivation of oxidative mitochondrial metabolism in epithelial cancer cells in situ: visualizing the therapeutic effects of metformin in tumor tissue. Cell Cycle.

[CR122] Wolf FI, Torsello A, Tedesco B, Fasanella S, Boninsegna A, D'Ascenzo M, Grassi C, Azzena GB, Cittadini A (2005). 50-Hz extremely low frequency electromagnetic fields enhance cell proliferation and DNA damage: possible involvement of a redox mechanism. Biochimica et Biophysica Acta (BBA)-Molecular Cell Research.

[CR123] Wong K-K, Engelman JA, Cantley LC (2010). Targeting the PI3K signaling pathway in cancer. Curr Opin Genet& development.

[CR124] Wood LD, Parsons DW, Sn J, Lin J, Sjoblom T, Leary RJ, Shen D, Boca SM, Barber T, Ptak J (2007). The genomic landscapes of human breast and colorectal cancers. Sci Signal.

[CR125] Woolston CM, Al-Attar A, Storr SJ, Ellis IO, Morgan DA, Martin SG (2011). Redox protein expression predicts radiotherapeutic response in early-stage invasive breast cancer patients. Int J Radiat Oncol Biol Phys.

[CR126] Wright DT, Cohn LA, Li H, Fischer B, Li CM, Adler KB (1994). Interactions of oxygen radicals with airway epithelium. Environmental health perspectives.

[CR127] Xiao C, Sharp JA, Kawahara M, Davalos AR, Difilippantonio MJ, Hu Y, Li W, Cao L, Buetow K, Ried T (2007). The XIST Noncoding RNA Functions Independently of BRCA1 in X Inactivation. Cell.

[CR128] Zaremba T, Olinski R (2010). Oxidative DNA damage–analysis and clinical significance. Postepy biochemistry.

[CR129] Zhang HJ, Zhao W, Venkataraman S, Robbins ME, Buettner GR, Kregel KC, Oberley LW (2002). Activation of matrix metalloproteinase-2 by overexpression of manganese superoxide dismutase in human breast cancer MCF-7 cells involves reactive oxygen species. J Biol Chem.

[CR130] Zhou L, Wang Y, Tian D, Yang J, Yang Y (2012). Decreased levels of nitric oxide production and nitric oxide synthase-2 expression are associated with the development and metastasis of hepatocellular carcinoma. Molecular medicine reports.

[CR131] Ziech D, Franco R, Georgakilas AG, Georgakila S, Malamou-Mitsi V, Schoneveld O, Pappa A, Panayiotidis MI (2010). The role of reactive oxygen species and oxidative stress in environmental carcinogenesis and biomarker development. Chemico-biological interactions.

[CR132] Z-y L, Yang Y, Ming M, Liu B (2011). Mitochondrial ROS generation for regulation of autophagic pathways in cancer. Biochem Biophys Res Commun.

