# Supplementary figures and images for: Involvement of free radicals in breast cancer
Source: Springerplus. 2013 Aug 27;2:404. doi: 10.1186/2193-1801-2-404 (PMC3765596; doi:10.1186/2193-1801-2-404)

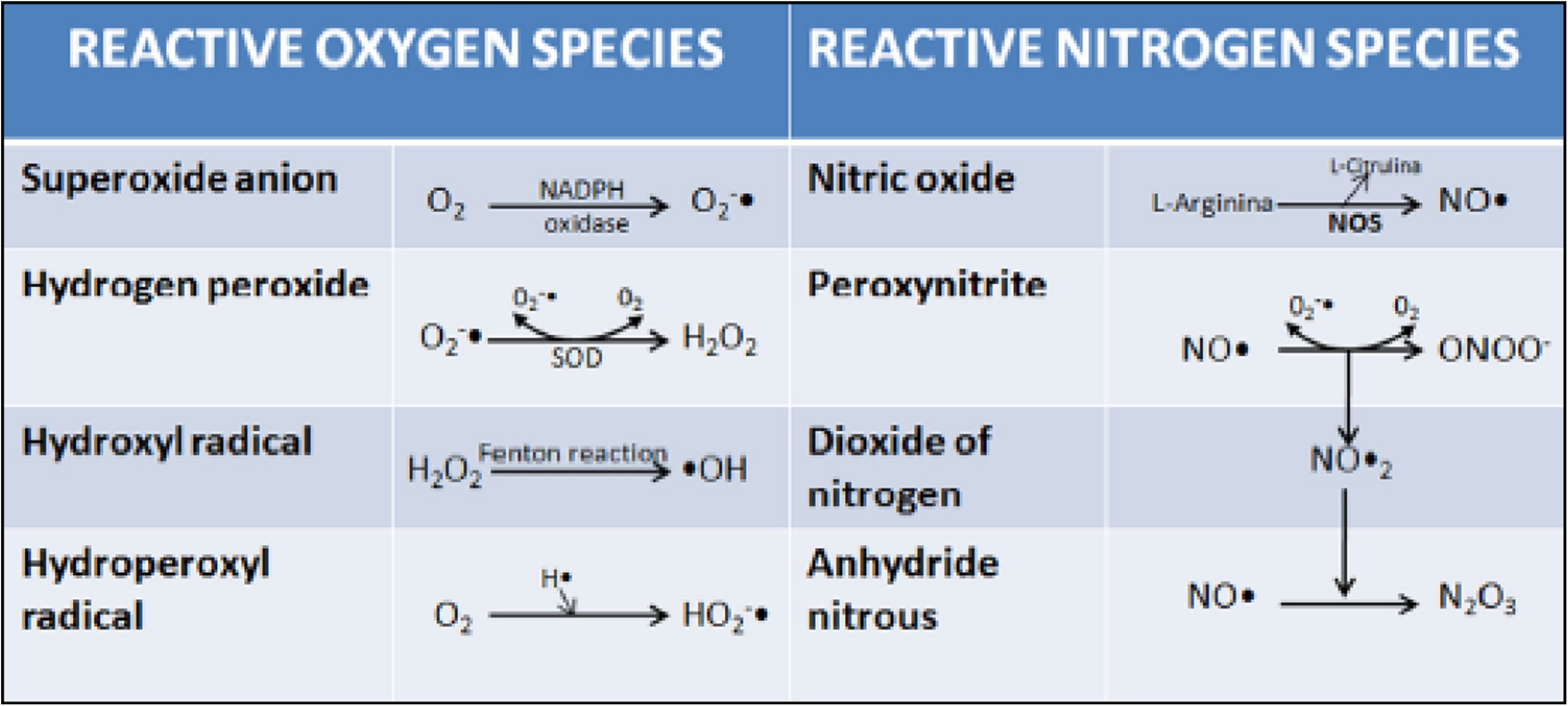

Supplement: Supplementary file 1 — Authors’ original file for figure 1 [file 40064_2013_475_MOESM1_ESM.tiff]

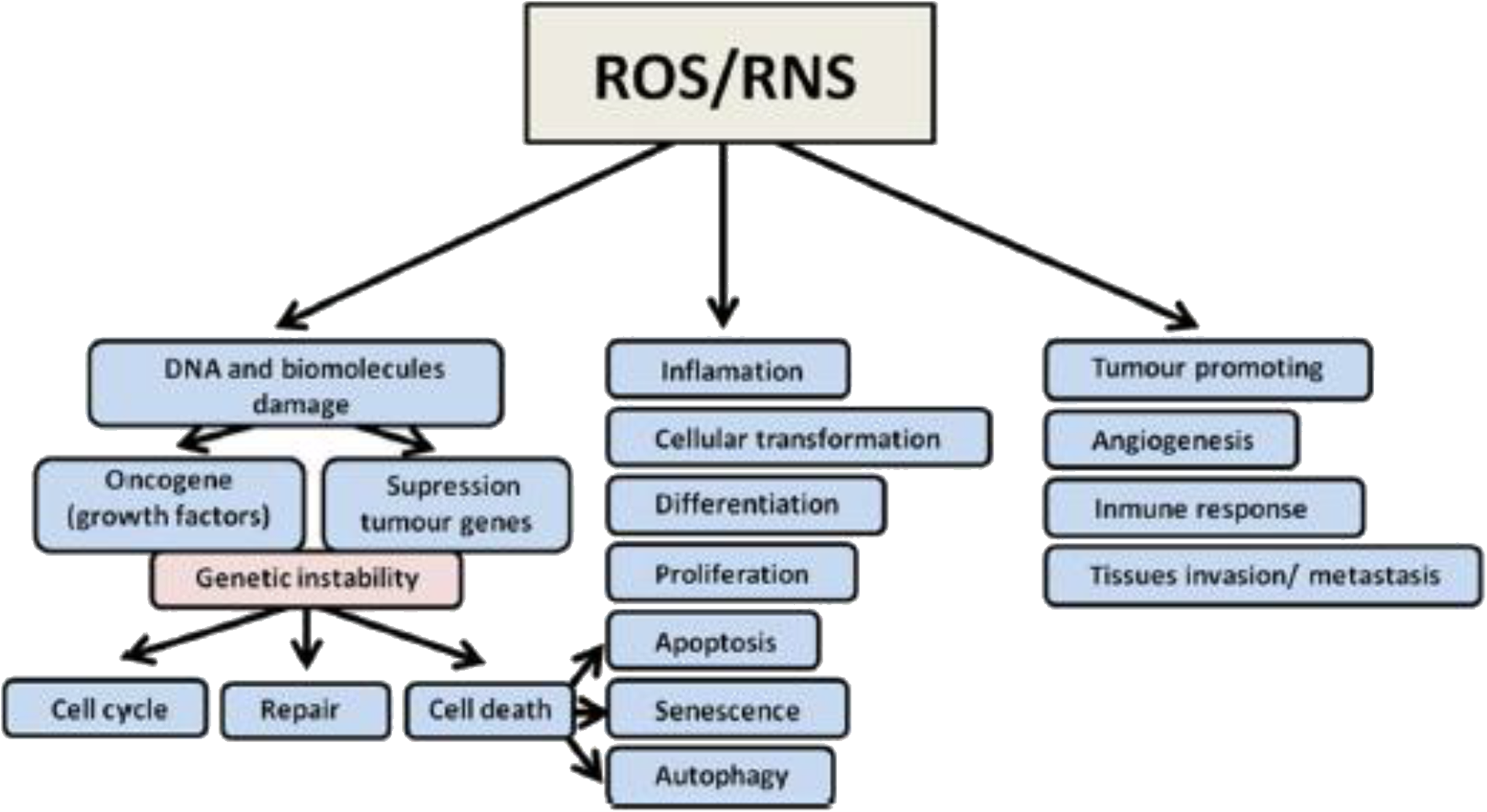

Supplement: Supplementary file 2 — Authors’ original file for figure 2 [file 40064_2013_475_MOESM2_ESM.tiff]
